# Fine-Needle Aspiration Biopsy of Hepatocellular Carcinoma and Related Hepatocellular Nodular Lesions in Cirrhosis: Controversies, Challenges, and Expectations

**DOI:** 10.4061/2011/587936

**Published:** 2011-06-30

**Authors:** Aileen Wee

**Affiliations:** Department of Pathology, Yong Loo Lin School of Medicine, National University of Singapore, National University Hospital, National University Health System, 5 Lower Kent Ridge Road, Singapore 119074

## Abstract

The role of hepatic fine-needle aspiration (FNA) biopsy has evolved. Advances in imaging modalities have obviated the need for tissue confirmation in most hepatocellular carcinomas (HCCs). There is risk of needle-tract seeding. Increasingly, small nodules are being detected on ultrasound surveillance of high-risk patients. Diagnostic challenges associated with cirrhosis include distinction of benign hepatocellular nodules, namely, large regenerative nodules and dysplastic nodules, from reactive hepatocytes; and distinction of well-differentiated HCCs from benign hepatocellular nodules. This paper will discuss (i) controversies regarding preoperative/pretransplantation FNA diagnosis of HCC, (ii) update of biological evolution, nomenclature, and histopathologic criteria for diagnosis of precancerous nodules and small HCCs, and (iii) algorithmic approach to FNA diagnosis of hepatocellular nodules. Optimal results depend on dedicated radiologist-cytopathologist team, on-site cytology service; combined cytohistologic approach, immunohistochemistry, and clinicopathologic correlation. Hepatic FNA is likely to be incorporated as a point of care as we move towards personalized medicine.

## 1. Introduction

The incidence of hepatocellular carcinoma (HCC) has risen as a result of increased global burden of chronic liver disease due to hepatitis B and C virus infections, aflatoxin B1, alcoholism, and nonalcoholic fatty liver disease/nonalcoholic steatohepatitis associated with the metabolic syndrome. It is now the sixth most common cancer worldwide and the third most common cause of cancer deaths [1]. The natural history in high-risk patients is the occurrence of dysplastic foci in a cirrhotic background from which precancerous dysplastic nodules may ensue with some transforming to become HCC [2, 3]. 

Much progress has been achieved over the years with regard to detection/screening, diagnosis, surveillance, and multimodal treatment approaches leading to improvement in prognosis of HCC [4, 5]. Surgery, and in particular liver transplantation, is considered the best option; but this is not widely available, and many patients still present with advanced disease. Increasing knowledge of molecular hepatocarcinogenesis has led to the advent of molecular targeted therapy [6]. Sorafenib, the antiproliferative and antiangiogenic multikinase inhibitor, has broadened the therapeutic horizon for advanced cases [5].

Early detection with appropriate therapy is still the optimal approach that offers the patient the best prognosis. High-risk patients undergo 6-monthly ultrasound screening and serum alpha-fetoprotein (AFP) assessment. Advances in dynamic imaging techniques have increased the accuracy of HCC diagnosis thus obviating the need for tissue confirmation [7–9]. The role of fine-needle aspiration biopsy (FNA) in the diagnosis of hepatocellular nodular lesions has evolved over the years. Smaller and smaller nodules are being detected on increasing surveillance of high-risk patients. Accurate tissue characterization of small well-differentiated hepatocellular nodular lesions (<2 cm) is very challenging and has significant therapeutic implications. 

There are two schools of thought with regards to preoperative/pretransplant FNA diagnosis of HCC. An update of the biological evolution and histopathologic criteria for the diagnosis of dysplastic nodules, small HCCs and “nodule-in-nodule” lesions is presented in tandem with clinically relevant nomenclature. An algorithmic approach to FNA diagnosis of HCC and hepatocellular nodular lesions is outlined. Current issues, controversies, challenges and future expectations are discussed. The focus is on hepatocellular nodular lesions associated with cirrhosis.

## 2. Fine-Needle Aspiration Biopsy from the Point of View of the Aspirator and Reader

*“Can indeterminate diagnoses of highly well-differentiated hepatocellular nodular lesions be reduced?”*

### 2.1. The Technique

There are various routes for performing FNA biopsy of the liver. Percutaneous (transabdominal) FNA biopsy performed under computed tomography (CT) or ultrasound (US) guidance has been adopted worldwide as a safe, efficient, and minimally invasive procedure for the diagnosis of focal liver lesions. It is useful for establishing inflammatory/infective conditions to ruling out or confirming malignancies, helping to distinguish primary from secondary lesions. This technique is especially advantageous in patients with advanced malignancies or who are poor surgical candidates. It can also be performed at laparoscopy or laparotomy under direct vision. The sensitivity and specificity of percutaneous FNA biopsy for detection of liver malignancy have been reported to be around 90% (range, 67%–100%) and 100%, respectively [9–11]. Sensitivity varies according to factors such as blind versus guided aspiration; number of passes; operator skill; size, location and consistency of the lesion; quality of smears; combined cytohistologic studies with ancillary testing; and reader expertise. The positive and negative predictive values and overall accuracy of FNA diagnosis for liver malignancy were reported in one large study to be 100%, 59.1%, and 92.4%, respectively [9]. False positives are rare. 

Endoscopic ultrasound-guided FNA (EUS-FNA) is the latest diagnostic and staging tool. It is safe, accurate, and versatile but highly operator dependent. EUS-FNA can access left lobe of liver, hilum, proximal right lobe, gallbladder, extrahepatic biliary system, and perihilar lymph nodes. It is especially useful for small and deep-seated left lobe lesions below CT/MRI resolution or not easily accessible to percutaneous FNA. As such, it enhances staging of liver metastases [12], and facilitates early detection of multifocal HCC in cirrhosis, thereby allowing for accurate assessment of number of lesions (intrahepatic staging of HCC) for transplantation eligibility purposes [13, 14]. Another advantage is concurrent sampling of pancreas and liver lesions, confirming primary and metastatic malignancy in one single diagnostic encounter. EUS-FNA has high sensitivity (82%–94%) and specificity (90%–100%) for malignancy [15–17]. However, as with percutaneous FNA, it gives a better diagnostic yield with metastases than with well-differentiated hepatocellular nodular lesions.

The needle size is between 20 to 22 gauge. Aspiration needles with cutting mechanism enable microbiopsy cores to be procured. In some instances, FNA samples can be more representative than wide-bore core needle biopsies because although the cores are broader they are shorter, and the core biopsy procedure is limited in flexibility and accessibility. With fine needles, multiple aspirations (up to four passes) can be safely performed in different directions, provided there are no contraindications.

### 2.2. Tissue Samples

The types of tissue samples obtained with FNA technique include smears, needle rinse samples, cell blocks, and microbiopsies from which core imprints can be made. Smears are air-dried and stained with Diff-Quik and/or May-Grünwald-Giemsa (MGG) as well as fixed in 95% alcohol and stained by the Papanicolaou method. To overcome paucity of histologic material, particulate material is quickly retrieved from the glass slides with a scalpel prior to staining and formalin-fixed for paraffin-embedded cell block preparation. The resultant histologic sections allow for study of architecture and for special stains and immunohistochemistry. Immunocytochemical studies may be necessary if only smears are available. 

Liquid-based cytology (LBC) of fine needle aspirates of HCC is not routinely accepted. Potential advantages include: (i) the aspirator can work alone putting the entire specimen into preservative solution, thus solving the problem of centralized pathology services receiving highly variable quality of sent-in smears; (ii) the smears are more representative as everything is collected on one slide with a portion stored in the permanent preservative solution for re-use when needed; (iii) multiple smears and cell block can be prepared for routine staining, immunostaining, and molecular tests; (iv) better preservation of cells; (v) shorter screening time as cells are concentrated in a 20-mm round area; and (vi) no interference from background elements [18–20]. Disadvantages include: (i) no on-site assessment of adequacy (unless split-sample method or double-aspiration protocol employed), (ii) no triage of specimens, (iii) no air-dried smears for Giemsa preparations, (iv) semiautomated processing requiring longer preparation time, (v) loss of information from removal of background elements, (vi) familiarity with cytologic artifacts and recognition of diagnostic pitfalls to avoid misinterpretations, and (vii) high cost. Under certain circumstances, smears of FNA material prepared by ThinPrep method may not be representative. It has been postulated that larger cell groups may settle out of the specimen so rapidly that they are not sucked onto the filter; or the groups may drop off the ThinPrep filter immediately after suction is removed and not be transferred to the prepared slide.

In the author's experience (unpublished data) with ThinPrep method for aspirates of HCC, the background tends to be devoid of red blood cells, debris, and bile; however, tumor diathesis, if excessive, is still discernible. The cellularity is much lower per slide than in conventional smears. The cell aggregates tend to be tighter, 3-dimensional, and decreased in size with partial/complete loss of cytoarchitecture, such as, arborizing trabecular structures, pseudoacinar rosettes, and peripheral and transgressing endothelium. Tumor cells appear generally smaller with nondescript/“angulated” shapes, denser cytoplasm, and frayed cell borders. Chromatin details, nucleoli, and intranuclear inclusions are more defined but difficult to appreciate in cells that are not monolayered. Cell blocks can be prepared from liquid-based specimens with reasonable quality; however, integrity of the trabecular-sinusoidal arrangement may be compromised. Immunocytochemistry can be successfully applied to thin-layer cytology slides [20]. However, just as in conventional smears, immunocytochemical interpretation can be an issue, especially in smears with low yield, dispersed cellularity, and obscured nuclear details. The general impression is that it is more challenging to decipher the cytologic characteristics of HCC in LBC material than on conventional smears. 

### 2.3. Diagnostic Challenges

The diagnostic challenges in FNA of focal liver lesions are (i) distinction of benign hepatocellular nodular lesions, namely, large regenerative nodule, dysplastic nodule, focal nodular hyperplasia, and hepatocellular adenoma from reactive hepatocytes; (ii) distinction of well-differentiated HCC from benign hepatocellular nodular lesions; (iii) distinction of poorly differentiated HCC from cholangiocarcinoma and metastatic carcinomas; (iv) determination of histogenesis of malignant tumor; and (v) determination of site of origin of malignant tumor [21]. At the well-differentiated end of the spectrum, cytohistologic features of malignancy are lacking whilst at the poorly differentiated end, clues to the cell lineage are found wanting (see Section 6).

### 2.4. Ways to Improve Accuracy Rates of FNA Biopsy Diagnoses

Optimal results are obtained with dedicated and experienced team comprising aspirator (radiologist- or endosonographer) and reader. An on-site cytology service with a cytopathologist or well-trained cytotechnologist is ideal [22, 23]. The service provides for rapid Diff-Quik-stained smears for assessment of sample adequacy, retrieval of particulate tissue for cell blocks, and triage of specimens for culture, flow cytometry and other ancillary tests, including molecular analysis. A combined cytohistologic approach is highly recommended with cell blocks and microbiopsies [24–26]. Immunohistochemistry has a great adjunctive role. The final diagnosis should be based on close clinicopathologic correlation. Resources should be provided to support training of cytotechnologists as well as for continuing professional development.

## 3. Fine Needle Aspiration Biopsy from the Point of View of the Hepato-Oncology Team

*“To perform or not to perform FNA biopsy for confirmation of diagnosis?”*

The last decade has seen much active debate over the role of FNA in the detection of HCC [7, 9, 27]. Advances in dynamic imaging techniques have increased the accuracy of HCC diagnosis. Recognition of the diagnostic value of contrast washout allowed for the refinement of the European Association for the Study of Liver 2000 Conference (EASL) guidelines and is reflected in the American Association for the Study of Liver Disease (AASLD) guidelines [7, 8]. Nodules larger than 2 cm occurring in cirrhotic livers are diagnosed as HCC if they show characteristic intense arterial profile with contrast washout in delayed venous phase on one dynamic imaging modality. Nodules measuring between 1 and 2 cm in cirrhotic livers require concurrence of two coincidental imaging modalities; otherwise, biopsy is recommended. The dilemma is whether to biopsy nodules <1 cm. The EASL guidelines recommend the “wait and see” policy with 3-monthly US surveillance. 

Those who oppose the performance of FNA biopsy cite the following reasons.

**(1) Advances in Dynamic Imaging Techniques are Sensitive and Accurate Enough for Establishing an HCC Diagnosis in most Nodules**

Advances in dynamic imaging modalities, such as contrast-enhanced US and dynamic magnetic resonance imaging (MRI), have yielded an accuracy, sensitivity, and specificity of 99.6%, 100%, and 98.9%, respectively, for the diagnosis of HCC [28]. The demonstration of an intensified contrast-enhanced arterial phase followed by delayed venous washout pattern is pathognomonic of HCC, thus, obviating the need for tissue confirmation. However, false positives do occur. According to Ghittoni et al, a diagnostic protocol based on imaging is like “a boat that leaks like a sieve.” Intrahepatic cholangiocarcinoma may occasionally have a hypervascular imaging pattern mimicking HCC [29]. False negatives are more likely to happen, especially with insufficient neoarterialization of small hepatocellular nodules. Many of these nodules may prove to be malignant in the long run.

**(2) Adopt the “Wait and See” Policy for Hepatocellular Nodules Measuring <1 cm**

The current EASL and AASLD guidelines are to biopsy nodules between 1 to 2 cm if no definitive diagnosis of HCC is reached on two coincidental imaging modalities, and to adopt the “wait and see” policy with more frequent US surveillance for nodules <1 cm [7, 8]. Small well-differentiated hepatocellular nodular lesions associated with cirrhosis range from large regenerative nodule, to low- and high-grade dysplastic nodules and small HCCs (early and progressed types) [30]. Studies on the biological evolution of small HCCs reveal that they tend to start off as dysplastic foci of hepatocytes exhibiting large cell or small cell change. Large cell change is detected in up to 81% of cirrhotic liver explants [31]. The incidence of small cell change in cirrhotic livers varies considerably ranging up to 50% in explants [31]. These abnormal foci may develop into low-grade (with large cell change) and high-grade (with small cell change) dysplastic nodules. The precancerous nature of small cell change is supported by high proliferative activity in the hepatocytes and morphologic resemblance to early HCC [2].

Malignant transformation may occur in dysplastic or even regenerative nodules giving rise to nodule-in-nodule lesions. High-grade dysplastic nodules become malignant in a third of cases [32]. Cytodiagnosis of these small nodules and the distinction of high-grade dysplastic nodule from early HCC are extremely challenging even with the aid of reticulin stain and novel immunohistochemical markers. Although the specificity of FNA for HCC is close to 100%, its negative predictive value is low. Hence, patients with negative biopsy findings should either undergo a second biopsy or enhanced surveillance [33].

It is the opinion of Caturelli et al that the prognostic implications of early diagnosis and treatment of HCC cannot justify this policy of “masterly inactivity” [27]. Firstly, it is incongruous to work on increasing detection of small nodules in high-risk patients and then recommend “masterly inactivity.” Secondly, more than half of the nodules <1 cm in cirrhotic livers prove to be HCCs (68%). Thirdly, US-guided FNA biopsies of hepatic nodules <1 cm in experienced hands with use of novel biomarkers and interpreted by expert reader yield correct diagnoses in about 90% of cases [34].

**(3) Risk of Needle-Tract Seeding**

The most contentious complication cited by detractors of the technique is the risk of needle-tract seeding turning a potentially operable case of HCC to a metastatic state [28]. Risk of implantation metastases after biopsy for malignancy in general is considered rare (0.003–0.009%); the incidence for HCC varies from 0.003% to 5% [34–36]. An overall incidence of 0.13% of HCC with soft tissue metastases was reported in one large study where a total of 18,227 person-times of FNA or percutaneous ethanol injection was performed on HCC patients [37]. The estimated rates of 18 and 22 G needle-induced seeding for HCC were 0.60% and 0.11%, respectively [37]. The interval between detected seeding and biopsy varies from several months to 3 years. Whilst some studies have shown that preoperative FNA has no statistically adverse effect on the operability, possibility of tumor spread, or long-term survival of HCC patients [38, 39], there are others which strongly maintain that pretransplant FNA diagnosis of HCC is not necessary [40, 41]. Seeding is usually noted with subcapsular tumors and those of high-grade malignancy; these tend to be tumors >2 cm. Hence, FNA of nodules 1 to 2 cm may be fairly free of seeding, and if these turn out to be HCC, they tend to be well differentiated.

**(4) Risk of Intraperitoneal Bleeding**

The major cause of death after percutaneous FNA is bleeding, mostly associated with severe cirrhosis with coagulopathy or large superficial tumors not covered by normal liver parenchyma [9]. A mortality rate of 0.018% was reported in a multi-institutional Italian series of 10,766 US-guided FNA biopsies [42]. Risk of bleeding is not a controversial contraindication.

**(5) Increased Risk of Tumor Recurrence and Posttransplantation Recurrence**

There is no clear evidence that, independent of tumor stage, patients who undergo FNA biopsy are at higher risk of tumor recurrence and posttransplantation recurrence due to biopsy-induced hematogenous dissemination of tumor cells [34, 36, 43]. It is possible that microinvasive tumor cell dissemination may have occurred prior to the procedure. Metachronous tumors may also arise from the residual oncogenic cirrhotic liver [4].

Those in favor of performing preoperative/pretransplant FNA cite the following reasons:

1. *Serum AFP Has Low Sensitivity*. Serum AFP is the most commonly used serum biomarker in conjunction with US in screening programs. It has low sensitivity (45%) [44], and the level has to be significantly elevated (400 ug/L) to be of any value as a screening tool. It is also usually not elevated in patients with small HCCs. Hence. It is prudent to perform FNA so as not to miss an early case of HCC. Development of novel serum surrogate markers, such as, glypican-3 (a membrane proteoglycan) could prove useful [45].
2. *To Allay Patient Anxiety Once a Liver Nodule Has Been Detected on Imaging*.
3. *To Cut Down on Costs of Long-Term Imaging Surveillance in the Long Run*.
4. *To Avoid a Futile Transplantation*. False-positive results from imaging techniques have occurred. The conundrum is to balance the risk of unnecessary surgery (2.5%) [28] against the risk of needle-tract seeding. The risk of seeding is overall lower than that of a futile transplantation with its attendant risks and life-long financial and medical issues.
5. *Eligibility for Liver Transplantation*. Liver transplantation provides the best overall outcome in that it removes the possibility of metachronous lesions in a cirrhotic liver and restores liver function. Pretransplantation biopsy is strongly recommended for transplantation listing, if HCC is the only reason for transplant in a compensated cirrhotic case [33]. In fact, the confirmation of HCC favorably alters the patient's candidacy for liver transplantation. A previous biopsy should not be considered a contraindication for transplantation. Although the specificity of FNA for HCC is close to 100%, its negative predictive value is low. Hence, patients with negative biopsy findings should either undergo a second biopsy or enhanced surveillance [33].
6. *For Immediate Institution of Anti-HCC Therapy When Lesion Is Still <2 cm*. The 5-year survival rate of early HCC is twice as high as that of progressed HCC [32]. It has been documented that 80% of small HCCs are already progressed and moderately differentiated with microinvasion of portal vein radicles (27%) and minute intrahepatic metastases or satellites (10%) at time of diagnosis [3]. Furthermore, 80% of patients with microinvasion and/or satellitosis suffer recurrence within the first 2 years of followup after surgery. In such cases, it would be prudent to perform FNA biopsy early, particularly in high-risk patients, so as not to miss that small window of opportunity for chance of cure.
7. *For Appropriate Therapy in Non-HCC Cases*. If resection appears to be the best option, biopsy may or may not be performed. When palliative treatment is planned, biopsy is recommended to avoid unnecessary/inappropriate treatment [46].
8. *Use of Coaxial Technique of Biopsy May Reduce Risk of Seeding*. A coaxial technique allows multiple samples to be obtained without repeated placement of the needle; thus, potentially reducing the risk of needle-tract seeding. This approach helps to reduce the number of inadequate biopsies and is preferred for small and distant lesions. It is highly recommended but has yet to be evaluated [47].
9. *FNA Biopsy as Point of Care*. Practices are likely to change. Prognostic factors to determine survival and recurrence rate include tumor size, localization, number of nodules, satellitosis, vascular invasion, and histologic grade. Tumor differentiation and vascular invasion show a strong correlation. Despite a potential bias due to sampling errors, FNA biopsy might help identify patients having well-differentiated HCCs with low risk of vascular invasion and good prognosis after transplantation.

Much research is being done in genomics and proteomics to determine the molecular signatures of HCCs. With the advent of molecular testing for better clinical tools for screening, diagnosis, surveillance, prediction of efficacy of treatment, monitoring of response, prognostication, and for rational targeted therapies, it is foreseen that FNA biopsy will be the most minimally invasive technique available to obtain samples of tumor and peritumoral tissues for molecular profiling [5, 6].

The issues that need to be addressed by hepatology teams were aptly put by Schölmerich and Schacherer [46], as: (i) how good is the technique and which is the preferred technical modality to perform a biopsy [efficacy of imaging-guided FNA], (ii) how dangerous is such a procedure and does it interfere with later treatment [complications and treatment options], and (iii) is a biopsy necessary and does it change the outcome [need for biopsy]? In the meantime, however, to biopsy or not to biopsy is a bedside decision to be made by the hepatology team, depending on the treatment options available. In developing/less developed countries where patients tend to present with more advanced disease, the practice of percutaneous US-guided FNA is still popular due to cost effectiveness, nonavailability of state-of-the-art imaging technologies, limited treatment options, and individual preference and expertise.

## 4. Nomenclature and Biological Evolution of Precancerous Lesions in Cirrhosis

Small hepatocellular nodules (≤2 cm) can occur in cirrhotic or noncirrhotic livers. They comprise large regenerative nodules, dysplastic nodules, and small HCCs. Focal nodular hyperplasia and hepatocellular adenoma are well-differentiated hepatocellular nodular lesions occurring in noncirrhotic livers and will not be discussed in this review. 

Recent studies on the biological evolution of hepatocellular nodules in cirrhosis have led to establishment of clinically relevant nomenclature for precancerous lesions and small HCCs [2, 6, 30]. Large cell change (“liver cell dysplasia”) is defined as hepatocytes displaying corresponding nuclear and cellular enlargement with preserved nuclear-cytoplasmic ratio. Small cell change (“small cell dysplasia”) is defined as hepatocytes exhibiting decreased cytoplasmic volume, cytoplasmic basophilia, mild nuclear pleomorphism, hyperchromasia, and increased nuclear-cytoplasmic ratio, giving an impression of nuclear crowding/increased cellular density. These groups of abnormal hepatocytes are referred to as dysplastic foci if they are <1 mm and dysplastic nodules if they are >1 mm in size. Dysplastic nodules can be low grade (large cell change) or high grade (small cell change). The precancerous nature of large cell change is still debatable. On the other hand, a high-grade dysplastic nodule indicates an increased risk for carcinoma development. 

Small HCCs (≤2 cm) are further categorized into early HCC (well-differentiated HCC with indistinct margins) and progressed HCC (well- to moderately differentiated HCC with distinct margins) [3, 30]. Distinctly nodular small HCCs usually contain well-developed unpaired arteries. Early HCCs may contain both portal tracts and unpaired arteries; similar features may be found in high-grade dysplastic nodules. Identification of high-grade dysplastic nodules and/or small HCCs should lead to treatment by local ablation, surgical resection or transplantation.

Awareness of the current morphologic criteria for the diagnosis of early HCC is helpful for the interpretation of small histologic samples and for choice of appropriate immunohistochemical panel. Early HCCs are characterized by the following histologic features [2, 30]: (i) increased cell density >2 times that of the surrounding tissue, with an increased nuclear-cytoplasmic ratio, (ii) irregular thin trabecular pattern (2 cells or more thick), (iii) pseudoglandular pattern, (iv) unpaired arteries, (v) diffuse fatty change, (vi) cytoplasmic basophilia or eosinophilia, (vii) sinusoidal capillarization, (viii) invasion of intratumoral portal tracts, and (ix) stromal invasion accompanied by lack of ductular reaction at the periphery of the nodules. Malignant transformation can occur within dysplastic or regenerative nodules, where any of the above features may be restricted to an expansile subnodule in the parent nodule (“nodule-in-nodule”). Under such circumstances, several passes in different directions during the FNA procedure may be required to overcome the focality of these proliferative foci. 

Fatty change, which can be observed in any hepatocellular nodular lesion, is reported in 40% of early HCC [6]. Prevalence of fatty change decreases along with increasing tumor size as neoarterialization increases. Sinusoidal capillarization is present diffusely in HCC and diffusely/focally in high-grade dysplastic nodule. Stromal invasion is the most helpful feature in differentiating early HCC from high-grade dysplastic nodule. Microinvasion of portal vein radicles is not expected in early HCC but may be encountered in progressed HCC. Microbiopsies allow for assessment of architecture and stromal invasion.

## 5. Clinical Implications of Molecular Subclassification of Hepatocellular Carcinoma

Hepatocellular carcinoma has a complex molecular pathogenesis and morphologic heterogeneity. Genomic and proteomic studies have helped elucidate the molecular signatures of HCCs. Recent studies have also identified molecular changes in HCC which are potential markers of early HCC, such as glypican-3. Furthermore, a specific gene-expression signature of the peritumoral liver tissue was found to correlate with late recurrence and survival [48]. A molecular subclassification will soon be added to the conventional morphologic classification; the purpose of which is to identify predictors of aggressive/indolent behavior or features associated with sensitivity/resistance to novel therapies [6].

The future role of pathologists will be to interrogate the tumor and the peritumoral tissues and to be able to interpret this situation for clinical use. Apart from the main objective of developing personalized molecular targeted anticancer treatment protocols, such an approach will also help to identify patients at high risk for recurrence/further development of cancer. This will allow for selective intensified clinical followup with possible chemopreventive strategies (personalized preventive medicine) [5]. The combination of multiple targeted agents is the next logical step in the treatment of HCC due to the strong rationale to inhibit as many signalling pathways as possible in hepatocarcinogenesis [49]. This is also useful for further treatment of sorafenib-resistant cases. What all this translates into on the practical front is that tissue samples will have to be procured from various parts of the tumor, and in inoperable cases, the FNA biopsy technique provides the best approach to date for tissue procurement.

## 6. Algorithmic Approach to FNA Diagnosis of HCC and Associated Hepatocellular Nodular Lesions

Well known for its heterogeneity, HCC has variants and mixed lesions that may mimic other tumors. On the other hand, metastases to the liver are by far commoner than primary liver cancers. Apart from cystic and inflammatory conditions, the major diagnostic issues in FNA of focal liver lesions are highlighted in Section 2 under “Diagnostic challenges” [21].

### 6.1. FNA Biopsy of Benign Hepatocellular Nodular Lesions

**Figure 1:**
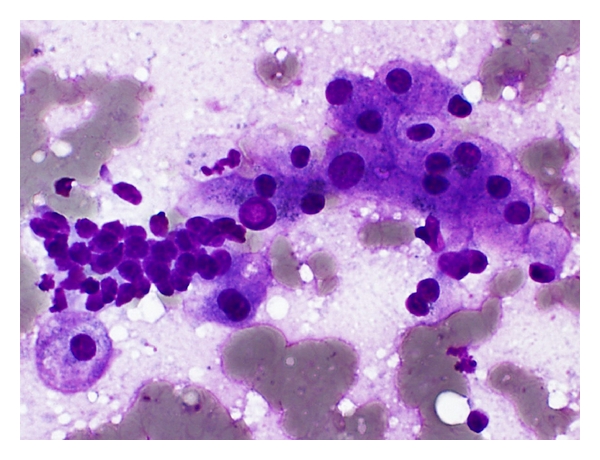
FNA of cirrhosis. Hyperplastic hepatocytes occur in 2-cell thick cords with closely adherent bile ductule. The polygonal hepatocytes exhibit cellular polymorphism with well-defined cell borders, ample granular cytoplasm, central round nucleus, and nuclear-cytoplasmic ratio of about 1/3. The ductule, indicative of hepatocellular-stromal interface restoration, consists of overlapping rows of small, ovoid, darkly-staining nuclei with nondiscernible cytoplasm. (Giemsa, ×400).

Aspirates of *cirrhotic nodules, including large regenerative nodules,* show a polymorphous population of cells, comprising hyperplastic hepatocytes, bile ductal epithelium, endothelial cells, and Kupffer cells, accompanied by features of regeneration and repair (Figure 1) [26, 50]. Polygonal hepatocytes have well-defined cell borders, ample granular cytoplasm, central round nuclei with well-defined nuclear membrane, granular chromatin, and distinct nucleolus. Nonneoplastic hepatocytes exhibit polymorphism, that is, sibling cells display variation in cell size and shape with corresponding variation in nuclear size. The nuclear-cytoplasmic ratio is about 1/3 (if one were to eyeball the nuclear and cell diameters). Hyperplastic hepatocytes show binucleate forms and appear as short 2-cell thick cords rather than singly. Fatty change, when present, is best appreciated in Giemsa preparations as intracytoplasmic vacuoles or as dispersed bubbles leaked from ruptured cells. Bile ductal epithelium appears as small flat clusters of cohesive uniform cells with minimal pale cytoplasm and bland, equidistant round to ovoid nuclei, and lacking nucleoli. Bile ductular epithelium is indicative of parenchymal-stromal interface restoration, and its recognition in lesional material is tantamount to confirming the benign status of the hepatocellular nodular lesion. Bile ductules appear as curved double-stranded rows of epithelial cells exhibiting ovoid darkly-staining overlapping nuclei with nuclear disarray, high nuclear-cytoplasmic ratio and barely visible cytoplasm, mimicking adenocarcinoma. Elongated endothelial and comma-shaped Kupffer cell nuclei can be identified amongst the hepatocytes. Inflammatory cells, comprising predominantly of lymphocytes, and stromal fragments may be encountered in the background. Focal nodular hyperplasia gives similar restoration features.

**Figure 2:**
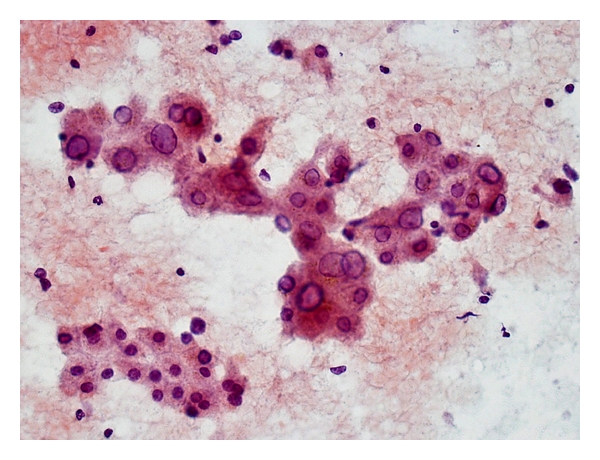
FNA of hepatocytes with large cell change. Two-cell-thick rows of hepatocytes show corresponding cellular and nuclear enlargement, maintaining normal nuclear-cytoplasmic ratio. Cellular polymorphism amongst sibling cells is clearly evident. Note intranuclear inclusions. Contrast with group of normal-sized hyperplastic hepatocytes (Papanicolaou, ×200).

Aspirates of *low-grade dysplastic nodules* contain hepatocytes exhibiting large cell change with no/minimal nuclear atypia and normal nuclear-cytoplasmic ratio (Figure 2). Hepatocytes from *high-grade-dysplastic nodules* are small and monotonous with subtle increase in nuclear-cytoplasmic ratio; the nuclear size is fairly similar to that of normal hepatocytes but there is less cytoplasm, thus imparting an impression of nuclear crowding [51]. Dysplastic hepatocytes generally occur singly or in 1- to 2-cell thick cords. Fatty change may be present. Bile ductal and ductular epithelium and stromal fragments may be evident in the background. It is difficult to distinguish high-grade dysplastic nodule and early HCC purely on cytologic grounds. 

### 6.2. FNA Biopsy of Classic Hepatocellular Carcinoma

Hepatocellular carcinomas are highly heterogeneous tumors with regard to differentiation, histologic patterns (trabecular-sinusoidal, pseudoacinar, and compact types), and cell morphology. As such, one should be fully cognizant of the challenges and limitations of FNA biopsy in the diagnosis of HCC. Several passes in different directions should be performed in large tumors to overcome diagnostic difficulties due to sampling bias. Accurate distinction of HCC and its variants from metastases is crucial for institution of appropriate therapy.

**Figure 3:**
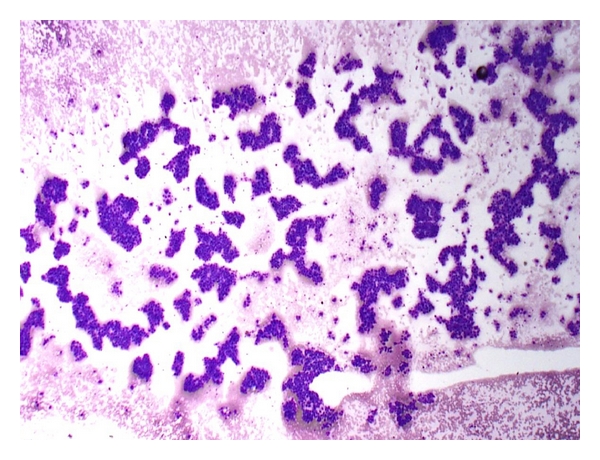
FNA of hepatocellular carcinoma. Low magnification view shows granular trails of irregularly shaped tumor aggregates (Giemsa, ×40).

**Figure 4:**
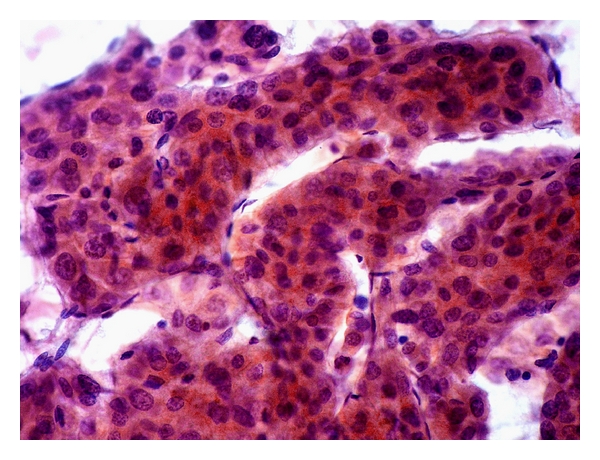
FNA of moderately differentiated hepatocellular carcinoma. Broad tongues of cohesive malignant hepatocytes wrapped by peripheral endothelium (Papanicolaou, ×400).

**Figure 5:**
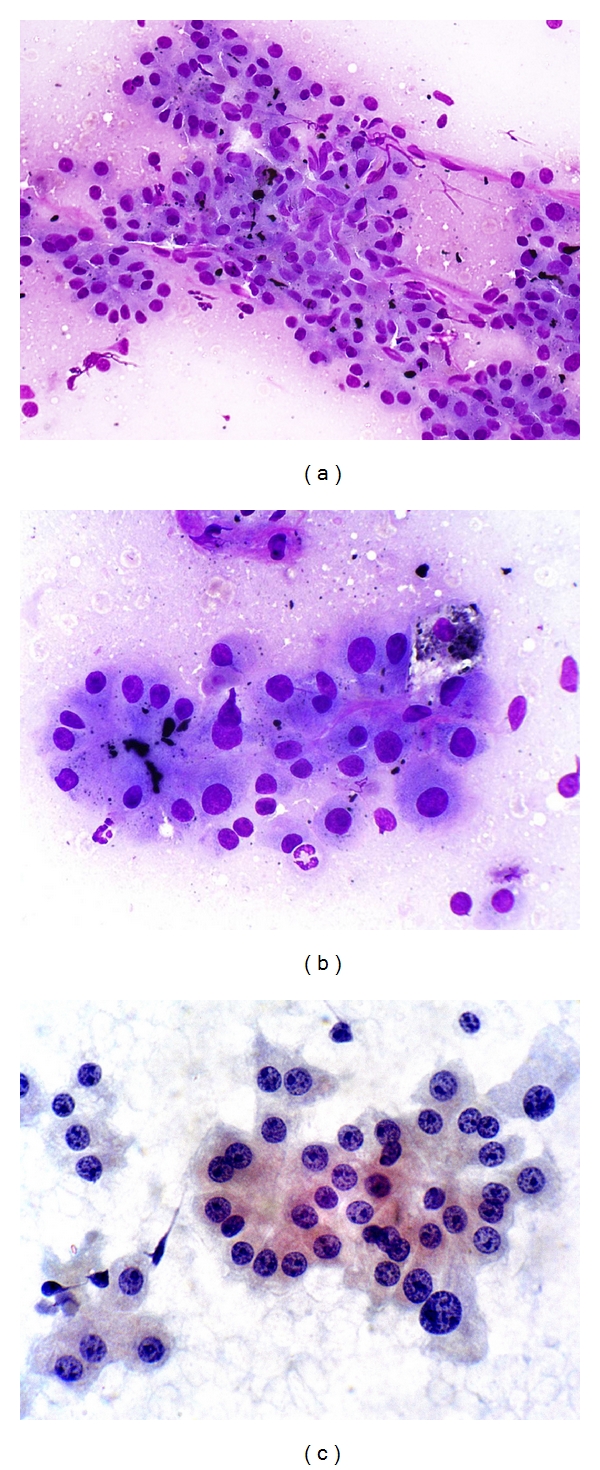
FNA of well-differentiated hepatocellular carcinoma. (a) Malignant hepatocytes exhibit pseudoacinar pattern with blackish bile plugs. Note transgressing endothelium with pink basement membrane material traversing tumor cells (Giemsa, ×200). (b) Rosette-like clusters of malignant hepatocytes form pseudoacini with blackish bile plugs within the dilated bile canaliculi. Some intracytoplasmic bile droplets are evident (Giemsa, ×400). (c) Similar pseudoacini are surrounded by malignant hepatocytes. The polygonal cells exhibit well-defined cell borders, ample granular cytoplasm, central round nucleus with well-defined nuclear membrane, distinct nucleolus and granular chromatin (Papanicolaou, ×400).

**Figure 6:**
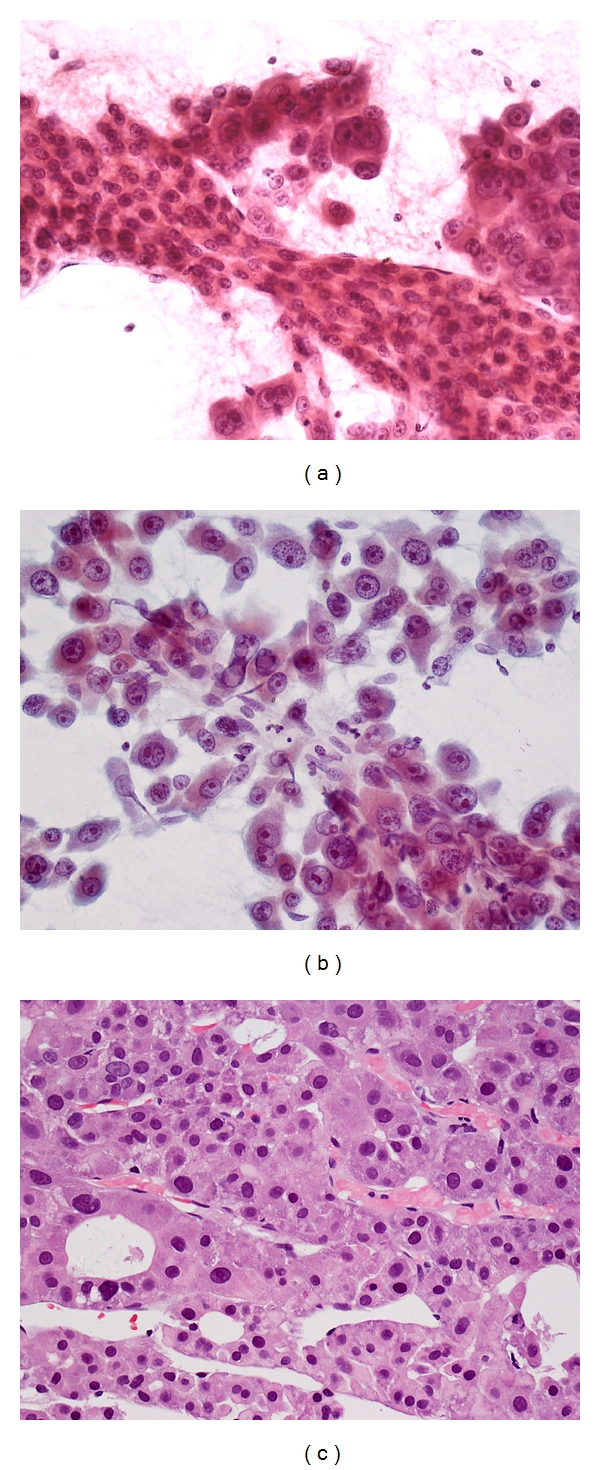
FNA of hepatocellular carcinoma. (a) Two populations of malignant hepatocytes are seen. One population forms a distinct broad trabecula with peripheral endothelium. The other tumor cells are bigger, more pleomorphic, and less cohesive (Papanicolaou, ×200). (b) The more pleomorphic cells appear discohesive but still retain recognizable hepatocytic characteristics. Note transgressing endothelium (Papanicolaou, ×400). (c) Cell block shows trabecular-sinusoidal and pseudoacinar patterns. (H&E, ×200).

**Figure 7:**
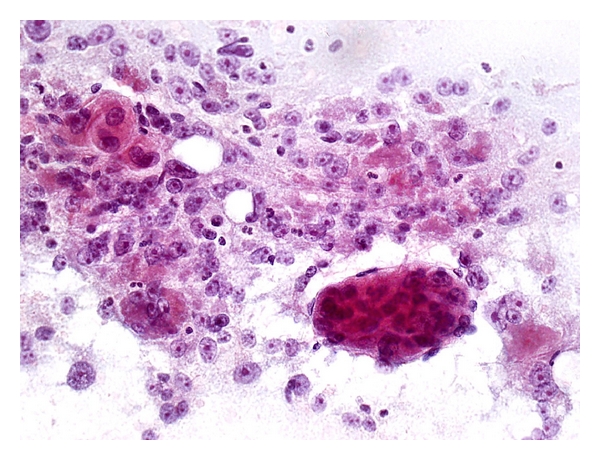
FNA of hepatocellular carcinoma. Atypical bare nuclei exhibit characteristic hepatocytic nuclear features. Mallory hyaline is seen as reddish clumpy intracytoplasmic material. Cross-section of a broad trabecula bordered by peripheral endothelium is evident (Papanicolaou, ×200).

**Figure 8:**
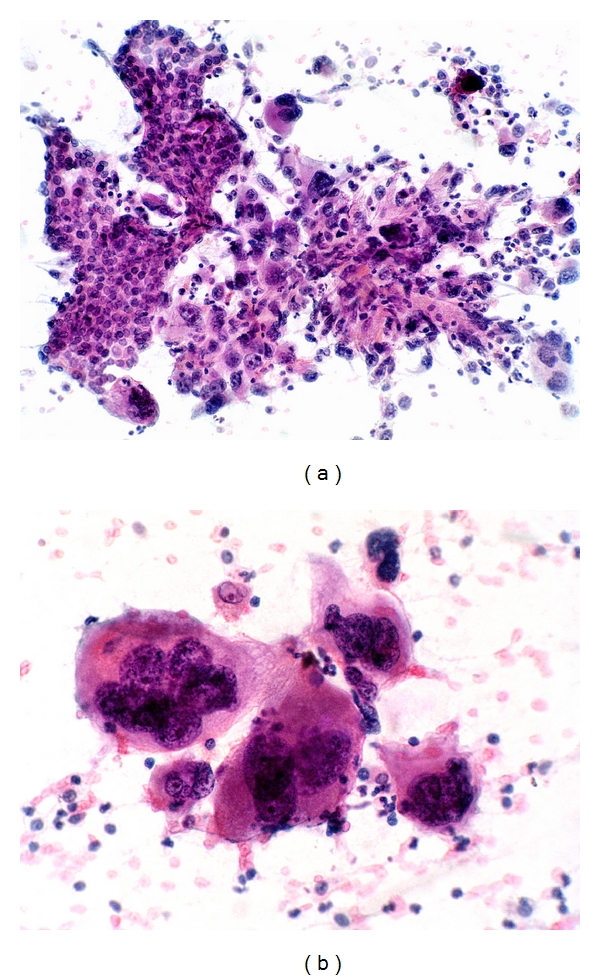
FNA of poorly differentiated hepatocellular carcinoma. (a) Two populations of tumor cells are seen. The better differentiated component is represented by a trabecula of malignant hepatocytes with peripheral endothelium. The other population consists of highly pleomorphic tumor cells with giant cells and bizarre nuclei; there is no apparent resemblance to hepatocytes (Papanicolaou, ×200). (b) Highly pleomorphic tumor giant cells exhibit multinucleation, bizarre nuclei, and hyperchromatism (Papanicolaou, ×400).

Cytologic features of HCC include [50]:

- *Hypercellular smears composed of trails of tumor cell clusters imparting a granular pattern of spread evident on gross inspection *(Figure 3).
- *Irregular arborizing, broad, tongue-like cords *(*>2 cells thick*) *of cohesive malignant hepatocytes* (Figure 4).
- *Peripheral endothelium wrapping broad cords *(Figure 4).
- *Transgressing endothelium running across larger aggregates* (Figure 5(a)): basement membrane material looking like pink “tramlines” (indicative of sinusoidal capillarization) is best seen in Giemsa preparations.
- *Cohesion is the rule: *tendency to dissociation is observed in highly well-differentiated HCC due to narrow cords; and in poorly differentiated HCC where there is virtually absent reticulin.
- *Pseudoacini containing bile or pale secretions *(Figure 5): polygonal neoplastic hepatocytes surround cystically dilated canaliculi.
- *Hepatocytic characteristics include polygonal cells with well-defined cell borders, ample dense granular cytoplasm, increased nuclear-cytoplasmic ratio *(>1/3)*, central round nucleus, well-delineated nuclear membrane, prominent nucleolus, and fine, irregularly granular chromatin*.* Mitoses increase with nuclear grade *(Figures 5(b) and 5(c)): cytologic features of malignancy are wanting at the well-differentiated HCC end whereas clues to hepatocytic histogenesis are lacking at the poorly differentiated end.
- *Tumor cells may be smaller, larger, or of the same size as nonneoplastic hepatocytes *(Figure 6): well-differentiated HCC cells tend to be conspicuous by their small size, monotony, subtle increase in nuclear-cytoplasmic ratio and nuclear crowding. Poorly differentiated HCC cells tend to be pleomorphic with thin nuclear membranes and irregular nuclear contours.
- *Atypical bare hepatocytic nuclei may abound* (Figure 7).
- *Multinucleated tumor giant cells may be of “osteoclastic” or pleomorphic type *(Figure 8): the former shows nuclear features akin to sibling tumor cells. Tumor giant cells may be found even in the lower grades of HCC. Their presence does not necessarily upgrade the tumor.
- *Bile may be present within tumor cells or in canaliculi or pseudoacini* (Figure 5): bile appears as greenish-black intracytoplasmic droplets, ropey intracanalicular strands and blobs within pseudoacini; best detected in Giemsa-stained smears.
- *Intracytoplasmic fat and glycogen vacuoles are common. Intracytoplasmic inclusions include hyaline, pale, and Mallory bodies *(Figure 7). *Intranuclear cytoplasmic inclusions are not specific. *
- *Bile duct epithelial cells, if present, are few and far apart. Background may be hemorrhagic and/or necrotic.*

Classic HCC is cytologically graded into well, moderately and poorly differentiated lesions based on nuclear grade.

**Figure 9:**
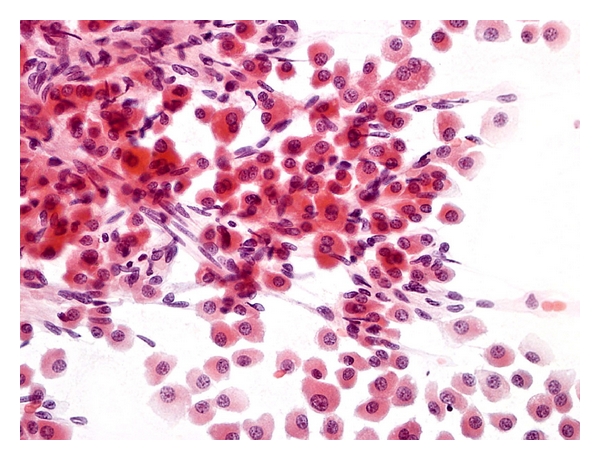
FNA of well-differentiated hepatocellular carcinoma. Small-sized malignant hepatocytes exhibit monotonous appearance with tendency to dissociation. The cells display well-defined cell borders, decreased dense cytoplasm, slightly eccentric nuclei, increased nuclear-cytoplasmic ratio, and impression of nuclear crowding. Transgressing endothelium abound (Papanicolaou, ×200).

*Well-differentiated HCC* (Figure 9): Tumor cells closely resemble nonneoplastic hepatocytes in size, shape and nuclear and nucleolar appearances. The nuclear-cytoplasmic ratio is slightly higher. Mitoses are exceptional.

*Moderately Differentiated HCC* **(** Figure 4). Tumor cells bear a resemblance to nonneoplastic hepatocytes. The nuclear-cytoplasmic ratio is moderately high, the round to ovoid nuclei show moderate degrees of pleomorphism, nucleoli are prominent, and mitoses are identifiable.

*Poorly Differentiated HCC *(Figure 8). There is poor resemblance to hepatocytes. Tumor cells exhibit marked pleomorphism, less cytoplasm, very high nuclear-cytoplasmic ratios, thinner nuclear membranes with irregular nuclear contours, hyperchromasia, and numerous mitoses. Nucleoli may be prominent or absent. Multinucleated tumor giant cells are easily identified.

**Cell Block/Microbiopsies**

The histologic diagnosis of HCC is based on cyto-architectural features, such as cell atypia, cell crowding, trabecular thickness, and microacini. Establishment of trabeculae ≥3 cells thick is one of the most helpful features in diagnosis of highly well-differentiated HCC. Gomori's silver stain for reticulin fibers is useful in distinguishing HCC from benign hepatic processes [52]. The reticulin framework is abnormal or deficient in HCC, and this coupled with the presence of broad cords accounts for their friability during the retrieval process for cell block preparation. Immunohistochemistry plays a helpful adjunctive role.

### 6.3. FNA Biopsy of Variants of Hepatocellular Carcinoma

Adequate representative sampling to achieve accurate cytodiagnosis of this heterogeneous malignancy will become more crucial when molecular subclassification of HCC is implemented for targeted therapy. The variations and variants of HCC include [53] the following.

**Figure 10:**
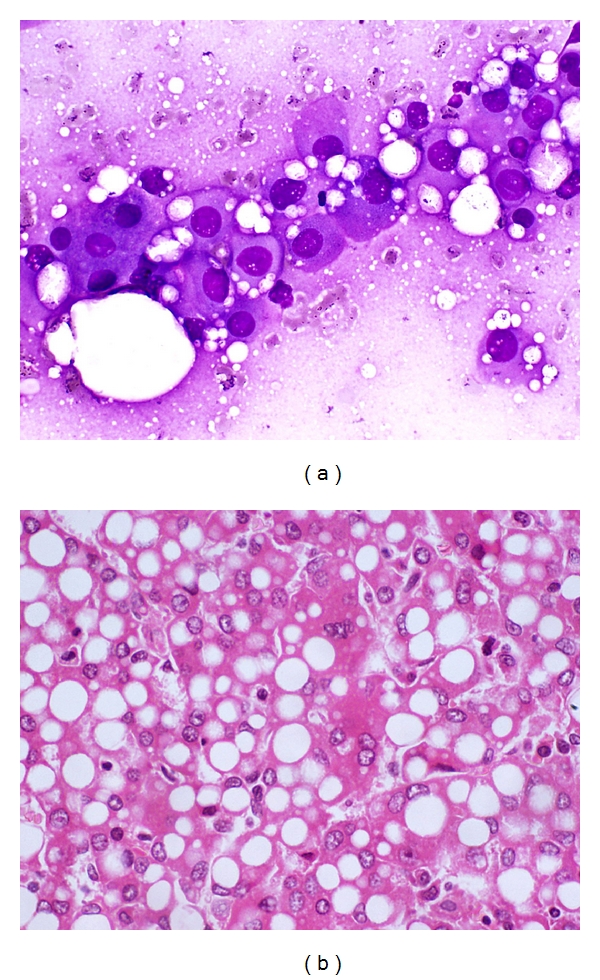
FNA of hepatocellular carcinoma with fatty change. (a) Malignant hepatocytes exhibit intracytoplasmic fat vacuoles of varying sizes. Lipid-containing bubbles are observed in the background (Giemsa, ×400). (b) Cell block shows fatty change in the tumor cells (H&E, ×200).

**Figure 11:**
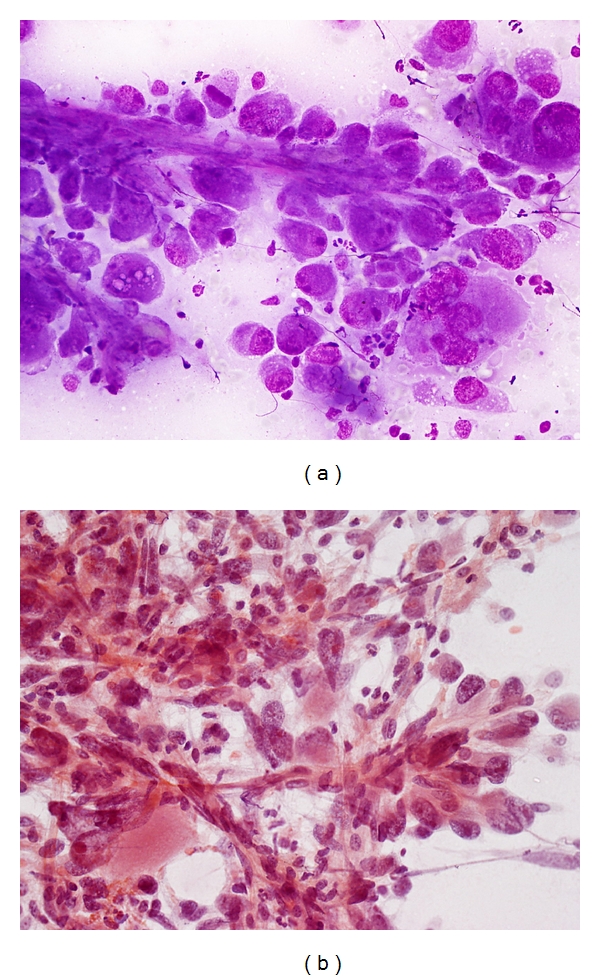
FNA of hepatocellular carcinoma with sarcomatoid change. (a) Loosely cohesive highly pleomorphic tumor cells exhibit sarcomatoid features. Note transgressing endothelium (Giemsa, ×400). (b) Spindle-shaped tumor cells and tumor giant cells bear no resemblance to hepatocytes. Transgressing endothelium abound (Papanicolaou, ×400).

- *HCC with Fatty Change* (Figure 10). Fatty change can occur in all sizes and grades of HCC. Highly well-differentiated HCC with fatty change can easily be overlooked for nonneoplastic hepatocytes from fatty liver or focal fatty change [50, 54].
- *HCC with Clear Cell Change.* Malignant hepatocytes with glycogen-laden cytoplasm display a pale/clear bubbly appearance, best appreciated in Giemsa preparations [55].
- *HCC with Small Cell Change.* The small tumor cells show scanty cytoplasm, round nuclei, high nuclear-cytoplasmic ratio, granular chromatin and small nucleolus. They mimic neuroendocrine tumors with a similar tendency to dissociation and microacinar formation [56, 57]; however, the “salt and pepper” chromatin of endocrine tumor cells is absent. Closer scrutiny of all available material may reveal more classic HCC features.
- *HCC with Pleomorphic Features.* The pleomorphic tumor cells are poorly differentiated with a tendency towards dissociation. Scattered multinucleated tumor giant cells and necrosis may be present.
- *HCC with Spindle Cell Features* (Figure 11). The pleomorphic spindle-shaped cells are indistinguishable cytologically from sarcomatous cells [58]. (see Sarcomatoid variant of HCC).
- *HCC with Giant Cell Features* (Figure 11). This pure variant is rare and has to be distinguished from giant cell sarcomas. Bizarre multinucleated giant cells with highly abnormal mitoses are present.
- *HCC with Biliary Differentiation.* Some HCCs may contain tubular spaces surrounded by columnar cells with basal palisading nuclei, favoring true acinar differentiation. HCCs may exhibit focal CK19 positivity—this may imply poorer prognosis [59]. Such HCCs have to be separated from the rare mixed type of HCC-CC.

#### 6.3.1. Special Types

- *HCC, Fibrolamellar Variant.* The tumor is characterized by monotonous population of discohesive, large polygonal cells with abundant oncocytic granular cytoplasm [60, 61]. Individual cells are about three times larger than normal hepatocytes, as are nuclear and nucleolar sizes.The nuclear-cytoplasmic ratio is generally <1/3. Intracytoplasmic pale and hyaline bodies are common. Presence of collagenous bands is a distinctive clue.
- *HCC, Scirrhous Type.* This type is uncommon and should not be confused with fibrolamellar carcinoma and cholangiocarcinoma.
- *Undifferentiated Type.* The tumor cells can be loosely cohesive or show tendency to dissociation. They are pleomorphic and nondescript with no cytologic clues to their hepatocytic histogenesis.
- *Lymphoepithelioma-Like Carcinoma.* It is a rare type of HCC with small pleomorphic tumor cells admixed with abundant lymphocytes. In some cases, the tumor cells are positive for Epstein-Barr virus [53].
- *Sarcomatoid HCC.* The purely sarcomatoid variant is rare; it is more often seen in conjunction with tumor giant cells. Extensive sampling may reveal areas of conventional HCC [58].

#### 6.3.2. Others

- *Combined Hepatocellular-Cholangiocarcinoma.* Although this combined (or mixed) tumor is characterized by an intimate admixture of HCC, cholangiocarcinoma, and a transitional component, not all components need be encountered in FNA material. HCC and cholangiocarcinoma are easily recognizable. However, transitional cells with features straddling HCC and adenocarcinoma may predominate [62, 63]. They may resemble malignant hepatocytes with trabecular arrangement but also exhibit acini with nuclear palisading. On the other hand, the transitional cells may display nuclear contour irregularities, indistinct nucleolus, and less granular cytoplasm. There may be difficulties distinguishing pseudoacini from true acini. Mucin may not be detected. The immunophenotype is often equivocal. A high index of suspicion is required for this cytodiagnosis.

**FNA Biopsy of Highly Well-Differentiated Hepatocellular Carcinoma**

**Figure 12:**
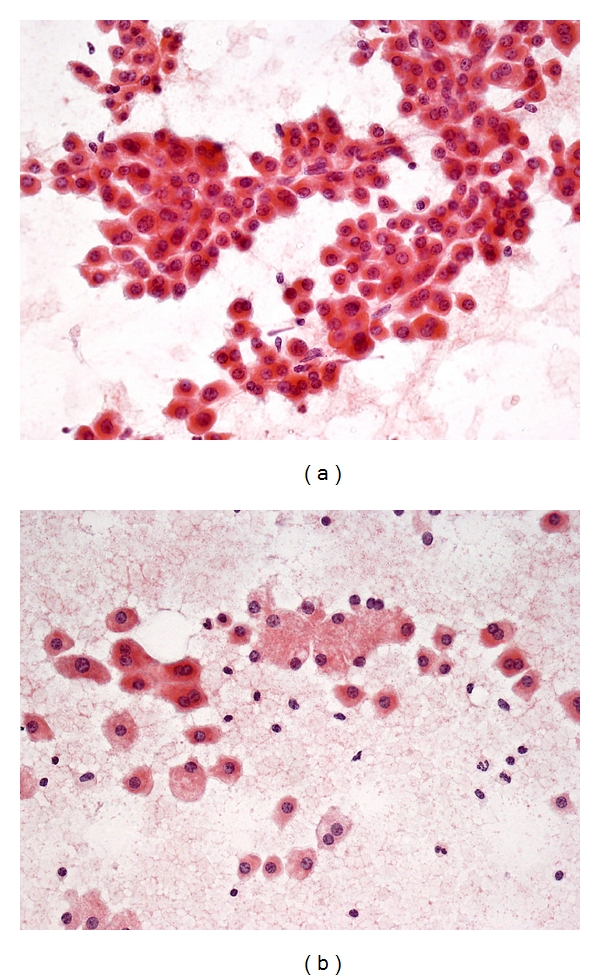
FNA of highly well-differentiated hepatocellular carcinoma from a “nodule-in-nodule” lesion. (a) Small and monotonous malignant hepatocytes exhibit decreased dense cytoplasm, central to slightly eccentric nuclei, increased nuclear-cytoplasmic ratio, and impression of nuclear crowding with closer inter-nuclear distances. Transgressing endothelium is present. Cytologic features are difficult to distinguish from those of a high-grade dysplastic nodule with small cell change. (Papanicolaou, ×200) (b) Two populations of dissociated hepatocytes are present. The malignant cells have dense eosinophilic cytoplasm with higher nuclear-cytoplasmic ratio. The nonneoplastic cells from the parent nodule have ample paler cytoplasm and normal nuclear-cytoplasmic ratio (Papanicolaou, ×200).

Diagnostic accuracy is certainly a challenge at this end of the spectrum and often indeterminate reports are rendered. Cytologic features predictive of HCC include increased nuclear-cytoplasmic ratio, cellular monomorphism, nuclear crowding, trabeculae >2 cells thick, atypical naked hepatocytic nuclei, and lack of bile duct cells [26, 51, 64, 65]. Cytologic parameters distinguishing highly well-differentiated HCC from cirrhosis include well-defined cell borders, scant cytoplasm, monotonous cytoplasm, thick cytoplasm, eccentric nuclei, and increased nuclear-cytoplasmic ratio [66]. An intimate admixture of neoplastic and nonneoplastic hepatocytes can be encountered in FNA of “nodule-in-nodule” lesions (Figure 12). Availability of cell blocks or microbiopsy material is very helpful for ancillary stains.

### 6.4. FNA Biopsy of Nonhepatocellular Mimics of Hepatocellular Carcinoma and Its Variants

#### 6.4.1. Benign Entities

**Hepatic Angiomyolipoma**

May consist of epithelioid and/or spindle cells with increased vascularity but without obvious adipocytes. A high index of suspicion and familiarity with this entity go a long way to avoiding the common pitfall of labeling the epithelioid variant with clear or oncocytic cells as HCC [67, 68]. Immunoreactivity of the tumor cells with HMB45 and desmin will help clinch the diagnosis.

**Inflammatory Pseudotumors**

Pose a distinct diagnostic pitfall clinically, radiologically, and cytomorphologically. Cytologic findings are highly variable. The pitfall of interest is mistaking reactive hepatocytes for HCC [69]. One should exercise extreme caution in making a diagnosis of malignancy in the face of concomitant inflammation.

#### 6.4.2. Malignant Entities

A practical approach to adopt when dealing with hepatic FNA of nonhepatocellular malignancies is to categorize them into the following cytomorphologic groups, namely, adenocarcinoma, squamous cell carcinoma, small round cell tumor, clear cell tumor, or malignancies characterized by pleomorphic, spindle, giant, or undifferentiated cells [21]. The initial cytologic assessment is crucial as it forms the impression upon which appropriate ancillary tests are ordered. Some of these cytomorphologic entities may occur de novo in the liver. At best, information gleaned from a precise cytodiagnosis can only favor a particular primary site. Close clinicopathologic correlation is mandatory.

**Figure 13:**
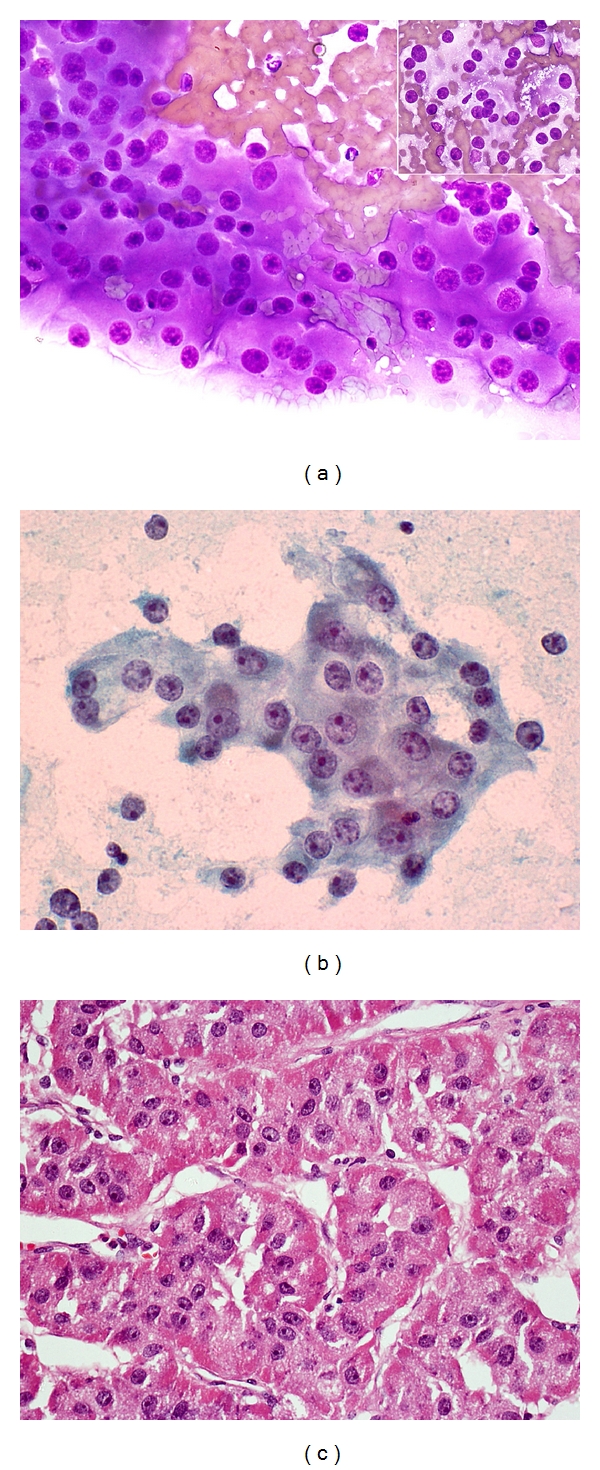
FNA of metastatic adrenocortical carcinoma to the liver. (a) Cohesive group of tumor cells with ample cytoplasm, central round nucleus, distinct nucleolus, and granular chromatin. (Giemsa, ×400). Inset: tumor cells contain fine lipid vacuoles in the cytoplasm (Giemsa, ×200). (b) Loosely cohesive tumor cells with nuclear features mimicking malignant hepatocytes (Papanicolaou, ×400). (c) Histology of adrenal tumor shows trabecular-sinusoidal pattern and polygonal cells with central round nucleus and nucleolus, mimicking HCC. The cytoplasm shows eosinophilic granularity (H&E, ×200).

Renal cell carcinoma, adrenocortical carcinoma, and melanoma are well-documented mimics of HCC (Figure 13). Metastatic and primary hepatic neuroendocrine tumors can occur [70]. The polygonal cell subtype of neuroendocrine tumor can mimic well-differentiated HCC whilst the small cell subtype can mimic small cell variant of HCC. Epithelioid and spindle variants of leiomyosarcoma/gastrointestinal stromal tumor may simulate HCC and its sarcomatoid variant, respectively [71, 72]. Rarely, extrahepatic AFP-producing hepatoid or nonhepatoid carcinomas, arising most commonly in the gastrointestinal tract, may occur. They have a proclivity for vascular permeation and liver metastases, giving rise to confusion with primary AFP-producing HCC [73].

## 7. Diagnostic Utility of Immunohistochemistry

An armamentarium of antibodies is available for the comparative immunohistochemical analysis of primary and metastatic tumors of the liver. A panel of immunostains has more discriminant value. Immunohistochemistry is preferred to immunocytochemistry. Careful light microscopic assessment of the histologic sections is important as judicious use of immunostains is imperative since material is limited. Double-staining protocols may help to optimize tissue usage. 

Kakar et al. outlined best practice guidelines for use of immunohistochemistry in the differential diagnosis of hepatic lesions under specific clinical scenarios [74]. Stepwise logistic regression analysis has shown that the panel of glypican-3, HepPar1, MOC-31, and CK7 is most useful in diagnosing and distinguishing HCC from metastatic adenocarcinoma on FNA material, with accuracy rates of 90.5 and 91.7%, respectively [75–78]. In the HCC group, glypican-3 was the most sensitive (81%), whereas HepPar1 (71.4%) and polyclonal carcinoembryonic antigen (pCEA) (50%) were less sensitive. In the metastatic adenocarcinoma group, MOC-31 was most sensitive (79.2) followed by CK7 (41.7%). 

**Figure 14:**
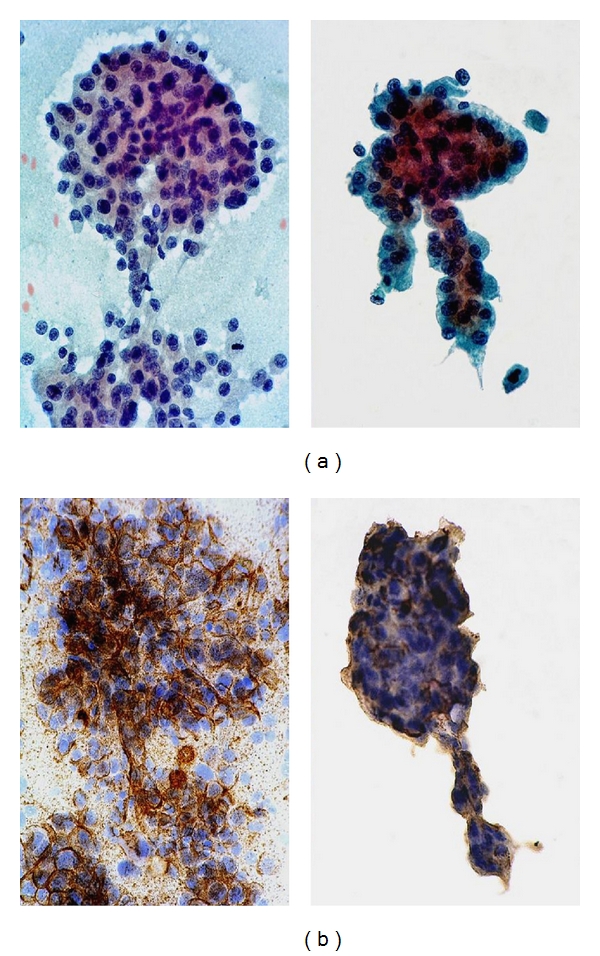
FNA of resected specimen of moderately differentiated hepatocellular carcinoma: conventional smears versus liquid-based cytology. (a) *Left panel*: Conventional smear shows larger and flatter aggregate of malignant hepatocytes. *Right panel*: ThinPrep smear shows a tighter, 3-dimensional cluster of malignant hepatocytes with trabeculae. The background is devoid of cells. Crisp nuclear details are better appreciated in monolayered cells. (Papanicolaou, ×400) (b)* Left panel*: Conventional smear shows malignant hepatocytes with bile canaliculi forming a delicate network of criss-crossing tubules highlighted by immunostaining with polyclonal CEA. *Right panel*: The canalicular network is more difficult to discern in the ThinPrep smear. (pCEA immunostain, ×400).

In the context of hepatocellular nodular lesions, the objectives are twofold [79]: (i) to prove hepatocellular histogenesis and (ii) to demonstrate the malignant status of the hepatocytes. For the former, the panel should include Hep Par 1 [80–82], TTF-1 [83, 84], and pCEA or CD10 to demonstrate canalicular formation (Figure 14) [85]. For the latter, the panel should include glypican-3, glutamine synthetase (a target protein of *β*-catenin), and heat shock protein 70 (a chaperone stress protein); two out of three positivity of these novel biomarkers are taken as indicative of HCC [86–90]. The demonstration of AFP positivity points towards a malignant tumor of hepatocellular origin provided nonseminomatous germ cell tumors and extrahepatic AFP-producing carcinomas have been excluded. Unfortunately, this tumor marker has such low sensitivity that it is no longer recommended as part of the panel [74]. If histologic material is available, use of CD34 highlights diffuse sinusoidal capillarization in HCC [74, 79]. CK19 can be used to demonstrate the absence of ductular reaction at the periphery of small HCCs, thereby confirming stromal invasion at the hepatocellular-stromal interface. The clinicopathologic and prognostic relevance of CK7 or 19 expression in HCC as indicative of possible progenitor cell origin for the tumor is still being studied [59]. Immunohistochemical results should always be interpreted in the larger context of the case.

## 8. The Future 

Our cytology role fits into the overall patient clinical pathway as FNA biopsy offers the potential immediacy of a diagnosis to the clinician who can then advise the patient and develop an appropriate next clinical step. The rapid turn-around time or at best reporting within 24 hours is ideal but for most practices this is not achievable with current resources. On-site cytology service provides immediate evaluation for adequacy and triage of specimens, which can be assessed by cytotechnologists rather than cytopathologists. The reduction in inadequate sample rates is important for overall cost effectiveness of the technique. 

The future will see a paradigm shift in the perception of the role of FNA in HCC. New trends in personalized molecular targeted therapy require better characterization and prediction of HCC behavior [48]. The FNA biopsy technique is still the most minimally invasive approach for the procurement of tumor and peritumoral tissue for molecular studies. We foresee that in the near future hepatic FNA is likely to become a point of care in the management of HCC patients, especially inoperable cases.

##  Conflict of Interests

The author declares that there is no conflict of interests.
